# Where did they not go? Considerations for generating pseudo-absences for telemetry-based habitat models

**DOI:** 10.1186/s40462-021-00240-2

**Published:** 2021-02-17

**Authors:** Elliott L. Hazen, Briana Abrahms, Stephanie Brodie, Gemma Carroll, Heather Welch, Steven J. Bograd

**Affiliations:** 1 NOAA Southwest Fisheries Science Center, Environmental Research Division, Monterey, CA USA; 2 Department of Ecology and Evolutionary Biology, University of California Santa Cruz, Santa Cruz, CA USA; 3 Institute of Marine Science, University of California Santa Cruz, Santa Cruz, CA USA; 4 Center for Ecosystem Sentinels, Department of Biology, University of Washington, Seattle, WA USA

## Abstract

**Background:**

Habitat suitability models give insight into the ecological drivers of species distributions and are increasingly common in management and conservation planning. Telemetry data can be used in habitat models to describe where animals were present, however this requires the use of presence-only modeling approaches or the generation of ‘pseudo-absences’ to simulate locations where animals did not go. To highlight considerations for generating pseudo-absences for telemetry-based habitat models, we explored how different methods of pseudo-absence generation affect model performance across species’ movement strategies, model types, and environments.

**Methods:**

We built habitat models for marine and terrestrial case studies, Northeast Pacific blue whales (*Balaenoptera musculus*) and African elephants (*Loxodonta africana*). We tested four pseudo-absence generation methods commonly used in telemetry-based habitat models: (1) *background* sampling; (2) sampling within a *buffer* zone around presence locations; (3) *correlated random walks* beginning at the tag release location; (4) *reverse correlated random walks* beginning at the last tag location. Habitat models were built using generalised linear mixed models, generalised additive mixed models, and boosted regression trees.

**Results:**

We found that the separation in environmental niche space between presences and pseudo-absences was the single most important driver of model explanatory power and predictive skill. This result was consistent across marine and terrestrial habitats, two species with vastly different movement syndromes, and three different model types. The best-performing pseudo-absence method depended on which created the greatest environmental separation: background sampling for blue whales and reverse correlated random walks for elephants. However, despite the fact that models with greater environmental separation performed better according to traditional predictive skill metrics, they did not always produce biologically realistic spatial predictions relative to known distributions.

**Conclusions:**

Habitat model performance may be positively biased in cases where pseudo-absences are sampled from environments that are dissimilar to presences. This emphasizes the need to carefully consider spatial extent of the sampling domain and environmental heterogeneity of pseudo-absence samples when developing habitat models, and highlights the importance of scrutinizing spatial predictions to ensure that habitat models are biologically realistic and fit for modeling objectives.

**Supplementary Information:**

The online version contains supplementary material available at 10.1186/s40462-021-00240-2.

## Background

Animal telemetry has revolutionized our understanding of animal movement and habitat use in both marine and terrestrial environments [29, 35]. Telemetry data have allowed for the exploration of behavioural and environmental drivers of animal space use, habitat selection, and migration [4, 39, 43, 49], and enabled the identification of important biological hotspots to inform conservation and management [14, 32, 55]. Animal telemetry data can also be used as inputs to habitat models (also known as ‘species distribution models’), to predict patterns of distribution or resource selection across space and time based on a species’ preference for particular characteristics of the environment [25]. However, a fundamental challenge of using telemetry data in habitat models is that they are presence-only, and thus cannot be used to infer environmental drivers in areas where animals were absent. To address this, a variety of techniques exist to generate data representing where animals could have gone but did not go (i.e. ‘pseudo-absences’, e.g. [9]). However, the relative performances of different pseudo-absence generation methods have not yet been assessed for telemetry-based habitat models. Furthermore, the literature lacks an evaluation of the relative utility of pseudo-absence methods between marine and terrestrial systems, where differences in the scales of habitat heterogeneity may influence model outcomes.

Approaches for generating pseudo-absences range from simple (e.g., background sampling, [46, 54]) to complex [e.g., biased sampling, [9, 41]]. Background sampling is the most commonly used approach, which involves randomly sampling the entire study area or habitat extent to produce absences that represent a broad range of characteristics [37, 38, 46]. While background sampling is the backbone of presence-only modeling techniques such as Maxent [54], it does not consider how animals actually move through space and treats all areas and habitats as being equally accessible. To address this issue, approaches that explicitly incorporate information on animal movement have been developed, such as buffer sampling (analogous to ‘step selection’ [7, 19, 60]. This approach treats habitat selection as a series of step-by-step decisions, with pseudo-absences randomly sampled within a predetermined step-length from each presence location. A third approach is to create pseudo-absences that have the same autocorrelation structure as actual tracks using correlated random walks (CRWs) [1, 30, 31, 42, 67]. CRWs recreate movement patterns using sampled step-lengths and turn angles from interpolated animal tracks, in order to realistically simulate the movement characteristics of study species. CRWs can also be generated in reverse (reverse CRWs) to control for biases generated by non-random animal tagging locations [53].

In order to highlight key considerations for generating pseudo-absences for habitat models built from telemetry data, the effects and biases of pseudo-absence generation methods need to be explored across species’ movement strategies, model types, and environments. Here we examine pseudo-absence generation methods using two mobile megafauna, the blue whale (*Balaenoptera musculus*) and African elephant (*Loxodonta africana*). These two species forage near the base of the food web, yet inhabit completely different physical environments and employ different movement strategies [2, 8, 61]. In the Northeast Pacific, blue whales undertake basin-scale migrations from breeding to foraging grounds, while in Etosha National Park, Namibia, elephants move nomadically within the park boundaries. For each species, we compare the effects of four different pseudo-absence generation techniques (background sampling, buffer sampling, CRW and reverse CRW) on habitat model performance. We compare results across three model types commonly applied to telemetry data (generalised linear mixed models, generalised additive mixed models and boosted regression trees) to test if the relative performance of different pseudo-absence generation methods was robust across different model types.

## Methods

### Species data

**Fig. 1:**
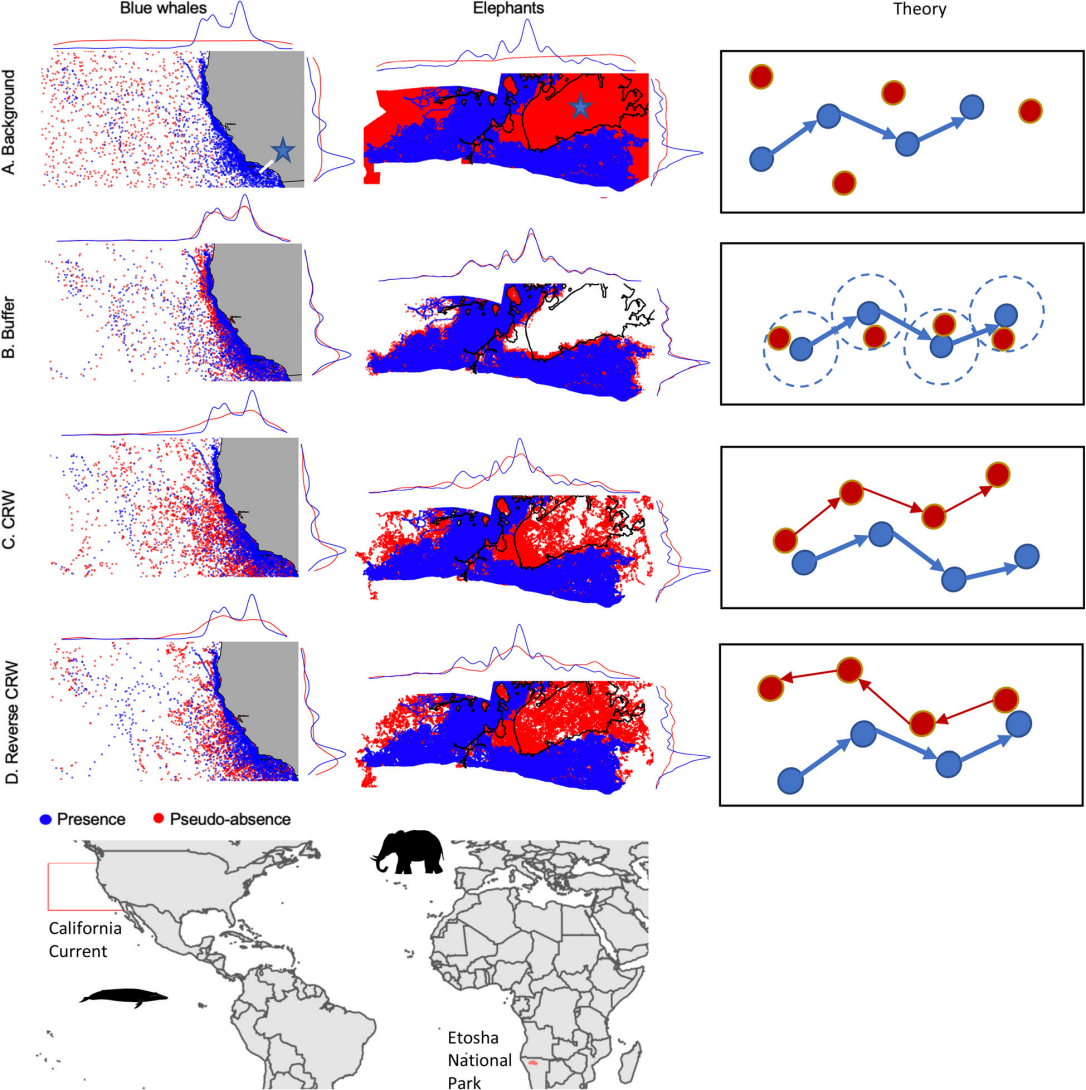
Presence data (blue points) and pseudo-absence data (red points) for the four pseudo-absence generation techniques **a** background, **b** buffer, **c** Correlated Random Walks (CRWs), **d** reverse CRW for blue whales (left), elephants (middle), and in theory (right). White represents areas unvisited by tagged individuals or simulated pseudo-absences. Density by latitude (top of panel) and longitude (right side of panel) highlights the difference in pseudo-absence sampling approach (red) from observed habitat using tracking data (blue). The Southern California Bight (top left) and salt pans (middle left) are indicated with blue stars. Study domains of the California Current, U.S. and Etosha National Park, Namibia are shown in the bottom two panels. In the right-most panels, the theory behind calculation of pseudo-absences for each approach is shown with blue being actual positions and red being simulated positions

We explored two previously published mega-vertebrate tracking datasets for Northeast Pacific blue whales and African elephants (Fig. 1). The blue whale data contained 10,664 daily locations in the eastern North Pacific, representing 104 ARGOS-tracked blue whales tracked between 1998 and 2009. This dataset has been studied extensively to identify critical habitat [36], understand patterns and drivers of migration [2, 8], and guide spatial management strategies [4, 30]. In this study, we examined foraging habitat selection by blue whales when resident in the central California Current System (CCS; 2,240,000 km^2^), excluding migratory behavior through Mexican waters and presumed breeding behavior in the southern end of their range. The elephant dataset contained 40,273 locations taken every 6 h from 14 GPS-collared elephants in Etosha National Park, Namibia (EtNP; 22,900 km^2^) between 2008 and 2014. These data have previously been used to explore animal movement syndromes [3] and drivers of habitat use [61].

### Environmental data

We selected six out of twelve potential environmental variables for blue whales that have previously been shown to be important drivers of habitat use during migration and foraging [4, 30, 52]: sea surface temperature, the spatial variability of sea surface temperature (an index of frontal activity), sea level anomaly, chlorophyll-a, oxygen concentration at 100 m depth, and bathymetry (Table S1). For elephants, we selected three variables that have been shown to most strongly influence elephant movement in the study area (Table S1, [61]): distance to the nearest road, multiannual mean normalized difference vegetation index (NDVI), and distance to the nearest water source. The two study systems, the CCS and EtNP, have vastly different patterns of environmental dynamism. The CCS has strong seasonal upwelling driving cool, productive nearshore waters [15], with offshore waters characterized by ephemeral features like fronts and eddies that can shift at daily to weekly timescales [20]. In contrast, EtNP experiences more gradual seasonal variation in temperature and rainfall [61]. Accordingly, the environmental variables selected for modelling mirror this dynamism: dynamic variables for the CCS were acquired at a daily or monthly resolution, whereas EtNP variables were either static or long-term averages (in the case of NDVI).

### Pseudo-absence types

We compared four methods of pseudo-absence generation that represent different assumptions about where animals could be distributed relative to observed tracks: ‘background sampling’, where random locations are sampled across the entire domain; ‘buffer sampling’, where random locations are sampled within a certain distance from each presence location; and ‘correlated random walks’ (CRW) and ‘reverse CRWs’, where tracks are simulated from given start or end points respectively, based on observed step lengths and turn angles. We outline each method below, and illustrate key concepts in Fig. 1.

Background sampling is designed to capture the full range of conditions under which species could be found, assuming they were distributed randomly across the environment. Habitat models are then used to contrast characteristics of preferred habitat where species are more likely to be observed, with this completely random distribution [21]. This approach is adapted from systematic survey design ([37] and references therein), where individual presences are not assumed to be autocorrelated [59]. Thus, even when applied to tracking data where each presence location depends on the one preceding it, background sampling of pseudoabsences incorporates no information or assumptions regarding characteristics of animal movement, such as distance traveled or direction of movement.

Buffer sampling for habitat modeling was originally used to minimize pseudo-absence overlap with presences, by sampling points outside a certain radius around each presence [33]. However, more recent approaches use buffers to restrict the sampling domain to areas accessible by the animal, by sampling from within a given radius around a presence [10, 24]. For tracking data, buffer size has been determined based on the mean or median step-length (e.g. distance traveled between two positions over a set time interval), irrespective of direction [34]. Resource selection functions use buffer sampling at each step to estimate the relative probability of selecting a specific parcel of habitat, relative to others that were equally accessible at that movement step [46].

CRWs and reverse CRWs sample the paired distribution of distance and turn angle from the empirical movement distributions in order to simulate realistic tracks (e.g. [1, 30]). CRWs have been used to create potential trajectories that animals could have taken based on measured movement parameters such as distance traveled and turning angle between consecutive locations [42]. CRWs have been implemented particularly when animals are wide ranging and can access areas far from the original tagging location [67]. In theory, CRWs offer the ability to create absences that best reflect the spatial and temporal auto-correlation of the actual tracks. Further, when there are implicit drivers of directionality or seasonality (e.g. movement away from competing colonies, or migration through less desirable habitat to reach more favorable habitat), entire CRW tracks can be selected that appropriately recreate important features of original tracks, such as the maximum displacement, or the mean angle of travel [30, 63]. Reverse CRWs have been introduced to address the issue of biases in tagging locations, recreating movement from the last known location and simulating backwards in time to the original tagging date [53].

### Pseudo-absence generation

We used a common sampling extent for all generation methods for each species based on the maximum extent of their tracks: for blue whales, a bounding box from 32° to 45° N and − 140° to − 115° W within the CCS; and for elephants the fenced boundary of EtNP (Fig. 1). For each pseudo-absence method, we generated a 1:1 ratio of pseudo-absences to presences to maintain consistency across models.

For background sampling, pseudo-absences were drawn randomly from within the domain for each species. For buffer sampling, we used the mode step length to create a radius of 100 km (whales) and 10 km (elephants) around each presence point, and randomly sampled one absence within each buffer zone. For CRWs, we randomly sampled a paired distance and turn angle from the observed distributions. Points were generated consecutively, starting from the locations where animals were tagged, until the number of pseudo-absences equaled the number of presences. The reverse CRW used the same approach but instead moved backwards in time from the last recorded position of the tag.

### Habitat modeling

We selected three commonly used statistical correlative models to test how model type influenced the relative performance of the pseudo-absence generation methods. We selected generalised linear mixed models (GLMMs), which are parametric and estimate linear species-environment relationships; generalised additive mixed models (GAMMs) which are semi-parametric and use smoothers to represent non-linear species-environment relationships; and boosted regression trees (BRTs) which are non-parametric and use boosting to determine optimal partitioning of variance. For both GLMMs and GAMMs, we used the *gamm* function in the ‘mgcv’ R package [64] and included individual tag identification as a random effect. For GAMMs, we used a thin-plate spline smoother with knots set to 5 per variable. BRTs were fit using the *gbm.fixed* function in the ‘dismo’ R package [23] with a learning rate of 0.005, a bag fraction of 0.75, tree complexity of 5, and 2000 trees (following [26]).

### Model performance

We evaluated model performance holistically across three dimensions: explanatory power, predictive skill, and biological realism. Explanatory power indicates a model’s ability to explain the variability in a given dataset, and was evaluated using % explained deviance (R^2^). Predictive skill indicates a model’s ability to correctly predict species presence or absence on novel data, and was evaluated with Area Under the Receiver Operating Characteristic Curve (AUC) and True Skill Statistic (TSS, [5]). As independent validation data do not exist at the scale of the original data, we tested predictive skill using three cross-validation approaches: the first used 100% of the data for both model training and testing. The second used randomly subsampled 75% of the data to train models, with the remaining 25% used to test models. Third, we also trained models on 11 of 12 months, and withheld a single month (twelve times) for testing for the dynamic blue whale models. As the terrestrial predictors for elephants were static or climatological averages, we were unable to test a temporal leave-one-out approach. We present the 100% training and testing results so that inferences were consistent across validation approaches.

Previous work has identified that habitat model performance will increase as environmental dissimilarity between presences and absences increases [45]. We explored this phenomenon by using density plots to qualitatively evaluate the environmental dissimilarity between presences and pseudo-absences generated by the four methods. Additionally, we quantified the statistical independence of the environmental niches of the presences and pseudo-absences for each variable and species using Bhattacharayya’s coefficient [13]. To determine the effect of environmental dissimilarity on model performance, we used linear regression to test relationships between Bhattacharyya’s coefficient and model predictive skill (AUC) for the three most important predictor variables for each species.

Finally, [62] recommended supplementing evaluations of model performance with evaluations of biological realism based on expert opinion and published literature. Following this advice, we qualitatively evaluated the ability of the models to predict realistic patterns of species distributions by assessing spatial prediction maps using expert knowledge. Specifically, we considered spatial predictions biologically realistic for blue whales if they predicted inshore habitat along the coast and reproduced the known blue whale hotspot in the Southern California Bight during summer months [12, 18, 36]; we considered spatial predictions biologically realistic for elephants if they avoided predictions in the large salt pan in the northeast corner of EtNP and preferred areas closer to roads, water, and fences [61]. We also quantified the ability of models to capture where blue whales and elephants are present and putatively absent by calculating mean predicted values at known presences and pseudo-absences, respectively.

## Results

### Spatial and environmental separation of pseudo-absences and presences

Blue whale presences were clustered adjacent to the California coastline, with highest densities in the Southern California Bight (Fig. 1). Elephant presences were clustered in the southern portion of EtNP, and no presences were located within the large salt pan in the northeast corner of the park (Fig. 1). There was similar spatial separation between pseudo-absences and presences across the four generation methods for both species (Fig. 1). Background sampling - which randomly sampled pseudo-absences across the study area - resulted in the greatest spatial contrast between pseudo-absences and presences, with pseudo-absences sampled in offshore regions of the CCS, and in the salt pan and northern extent of EtNP (Fig. 1). Buffer sampling - which sampled pseudo-absences within 100 km and 10 km of blue whale and elephant presences, respectively, resulted in the lowest spatial contrast between pseudo-absences and presences, while CRW and reverse CRW resulted in intermediate spatial contrast (Fig. 1).

**Fig. 2:**
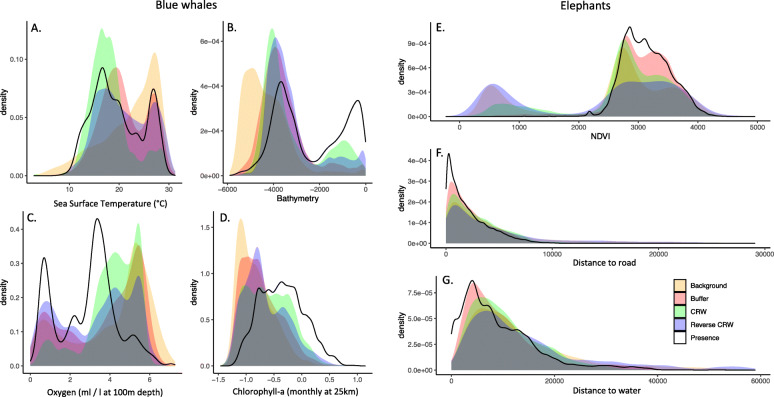
Degree of environmental separation for key predictor variables between presences (black line) and each pseudo-absence generation technique (colors) for blue whales (**a**-**d**), and elephants (**e**-**g**). Grey shading represents overlap across all techniques

The separation of environmental variables between presence and pseudo-absence locations were similar to the spatial contrasts among pseudo-absence generation methods (Fig. 2). For blue whales, background sampling had the greatest environmental separation between presences and pseudo-absences for all variables, largely due to the preference of tracked animals for the nearshore 200 m depth contour and the strong onshore-offshore environmental gradients that were sampled by the pseudo-absences (Fig. 2a-d). For example, sea surface temperature had a single peak at 28 °C for background sampling, compared to double peaks around 28 °C and 16 °C in the presence data, CRW, reverse CRW, and buffer sampling (Fig. 2a). All pseudo-absence methods sampled deeper, more oxygenated waters with lower chlorophyll concentrations compared to the blue whale presences (Fig. 2b-d). The elephants showed less environmental separation between pseudo-absences and presences compared to blue whales, and fewer differences in separation among pseudo-absence methods (Fig. 2e-g). For elephants, buffer sampling resulted in the greatest environmental overlap between pseudo-absences and presences for the three predictor variables, whereas reverse CRW sampling had the lowest overlap with presences. Pseudo-absence methods generally sampled areas that were further from roads and water, and with lower NDVI values compared to where elephants were present (Fig. 2e-g). For both species, habitat model response curves highlighted how unique the environmental data range of background sampling was compared to the other pseudo-absence methods (Fig. S1).

### Model performance

Blue whale model performance was strongly driven by pseudo-absence type, with models built using background sampling having the best explanatory power, predictive skill, and ability to capture where blue whales are present (Table 1). CRWs were best able to capture where blue whales were absent (mean prediction at pseudo-absences). In contrast, elephant model performance was predominantly influenced by model type, with BRTs having the best explanatory power, predictive skill, and ability to capture where elephants were absent regardless of pseudo-absence type. This pattern of BRTs performing best was also apparent in blue whales, but to a lesser extent due to the large effect of pseudo-absence type (Table 1). Following BRTs, GAMMs outperformed GLMMs in terms of explanatory power and predictive skill for both species.

**Table 1:**
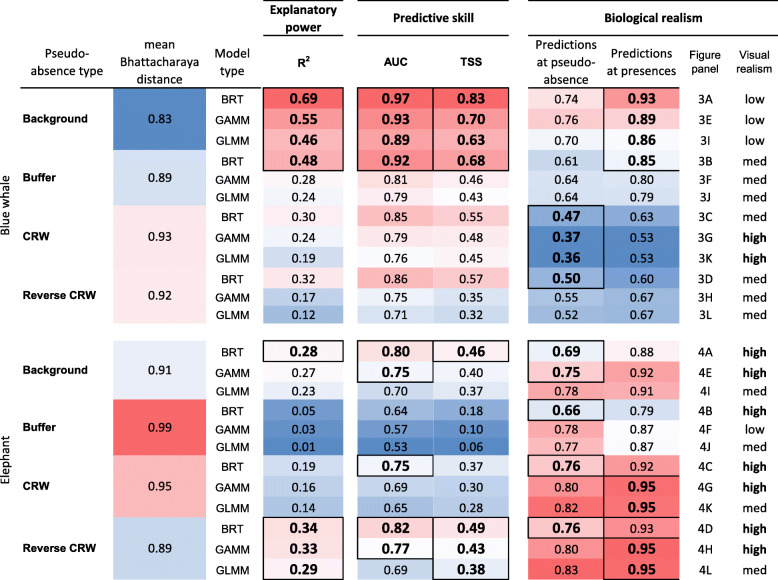
Summary of model predictive skill statistics (R2, AUC, TSS) for blue whale and elephant habitat models, each model type, and each pseudo-absence generation technique. Biological realism was assessed using the predictions at simulated absences and true presences, with visual realism assessed by the full suite of authors based on skill within the Southern California Bight (blue whales) and Etosha salt pan (elephants). Figure panel is also included for Fig. 4 (blue whales) and 5 (elephants) to aid cross-referencing. The best performing model using 100% test and training is shown in red with the worst shown in blue. For R2, AUC, TSS, and Predictions at presences, high values indicate better performance. For Predictions at pseudo-absence, values closer to 0 indicate better performance. Bold values are the top 4 performing models in each category, with blue backgrounds representing the best performing in that category and red representing worse

**Fig. 3:**
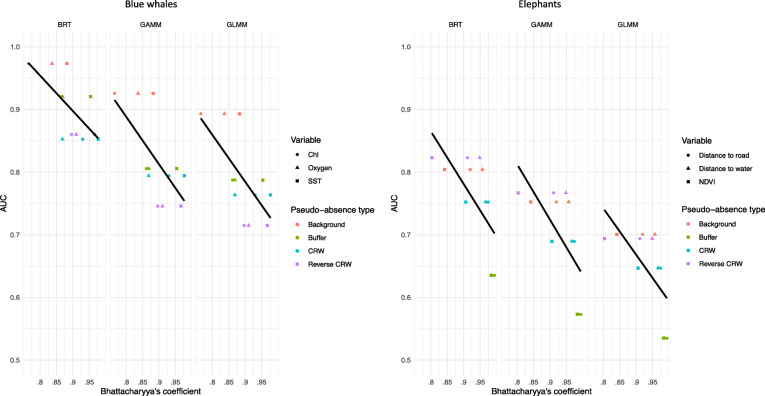
Relationship between model predictive skill (AUC; Area Under the Receiver Operating Characteristic Curve) and environmental separation between presences and pseudo-absences (Bhattacharyya’s coefficient) for blue whales (upper) and elephants (lower). Bhattacharyya’s coefficient was calculated for key environmental covariates (symbols). Sub-panels for each model type (BRT, GAMM, GLMM) are shown, with colors indicating pseudo-absence generation technique. The lines represent linear regression between the AUC value and the Bhattacharyya’s coefficient independent of pseudo-absence type and variable

Environmental similarity between presences and pseudo-absences (Bhattacharyya’s coefficient) had a significant negative relationship (*p* < 0.05) with model predictive skill (AUC) for each model type and species (Fig. 3). That is, as the environments sampled by pseudo-absences became more similar to presence locations, model performance decreased. This pattern was also reflected in the relationship between Bhattacharyya’s coefficient and both TSS and R^2^ values (Table 1). The lowest Bhattacharyya’s coefficient (highest environmental separation) was found in blue whale background sampling, which also had the highest R^2^, AUC, and TSS values across all models and both species. Conversely, the highest Bhattacharyya’s coefficient (lowest environmental separation) was found in the elephant buffer sampling, which also had the lowest R^2^, AUC, and TSS values across all models and species (Table 1, Tables S1, S2). These results provide evidence that model explanatory power and predictive skill is strongly related to environmental separation between presences and absences, regardless of species or habitat model type.

**Fig. 4:**
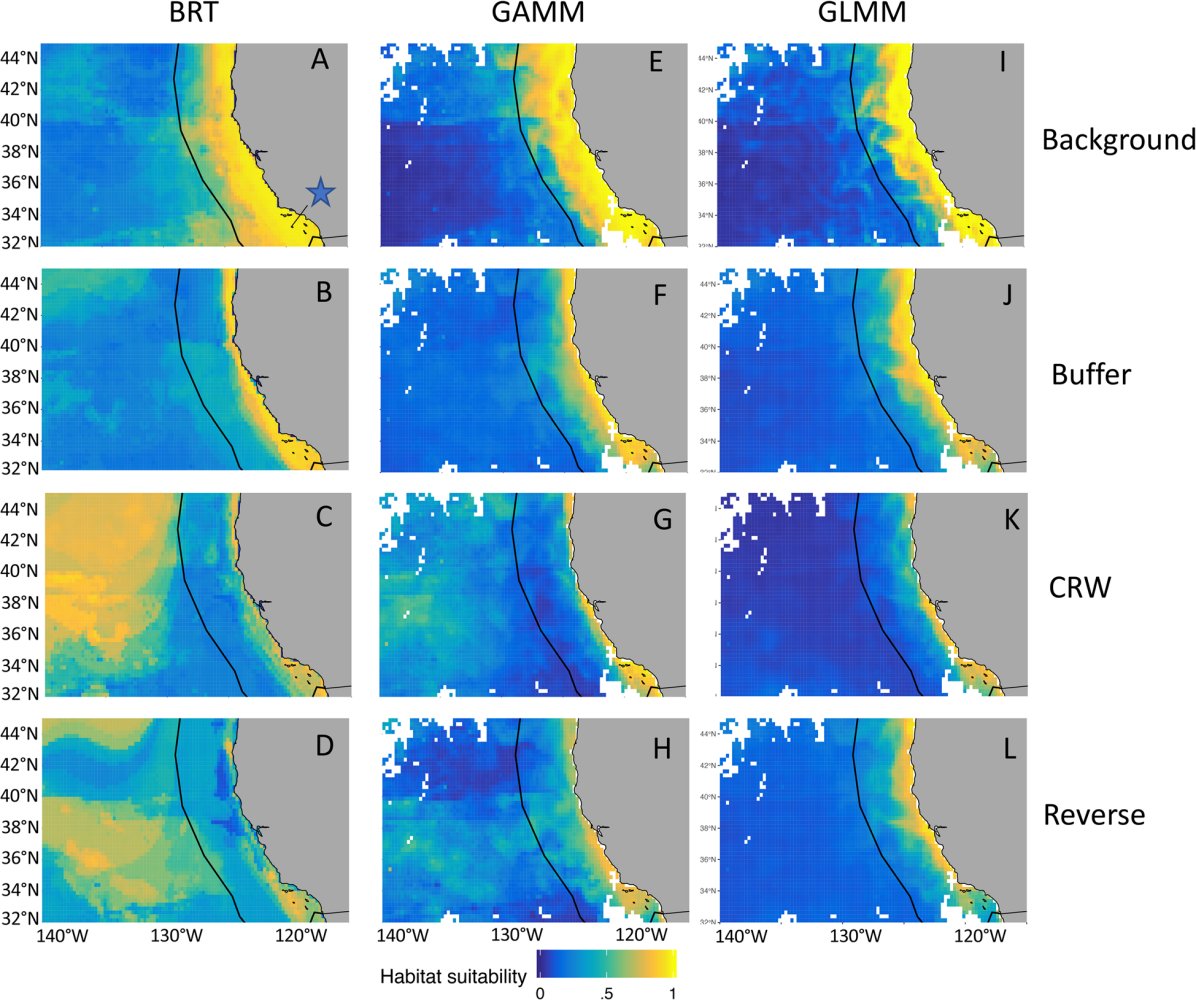
Effect of pseudo-absence generation type for BRT (**a**-**d**, four panels on left), GAMM (**e**-**h**), and GLMM models (**i**-**l**) and model type using background sampling (**a**, **e**, **i** - top three panels), buffer sampling (**b**, **f**, **j**), CRW sampling (**c**, **g**, **k**), and reverse CRW sampling (**d**, **h**, **l**) on blue whale model predictions for a given day, August 1st, 2006. Yellow indicates high habitat suitability while blue is low habitat suitability. GLMMs and GAMMs have white pixels where there were missing predictor variables (e.g. due to cloud cover) for the day. The blue star in panel A is pointing to the Southern California Bight

**Fig. 5:**
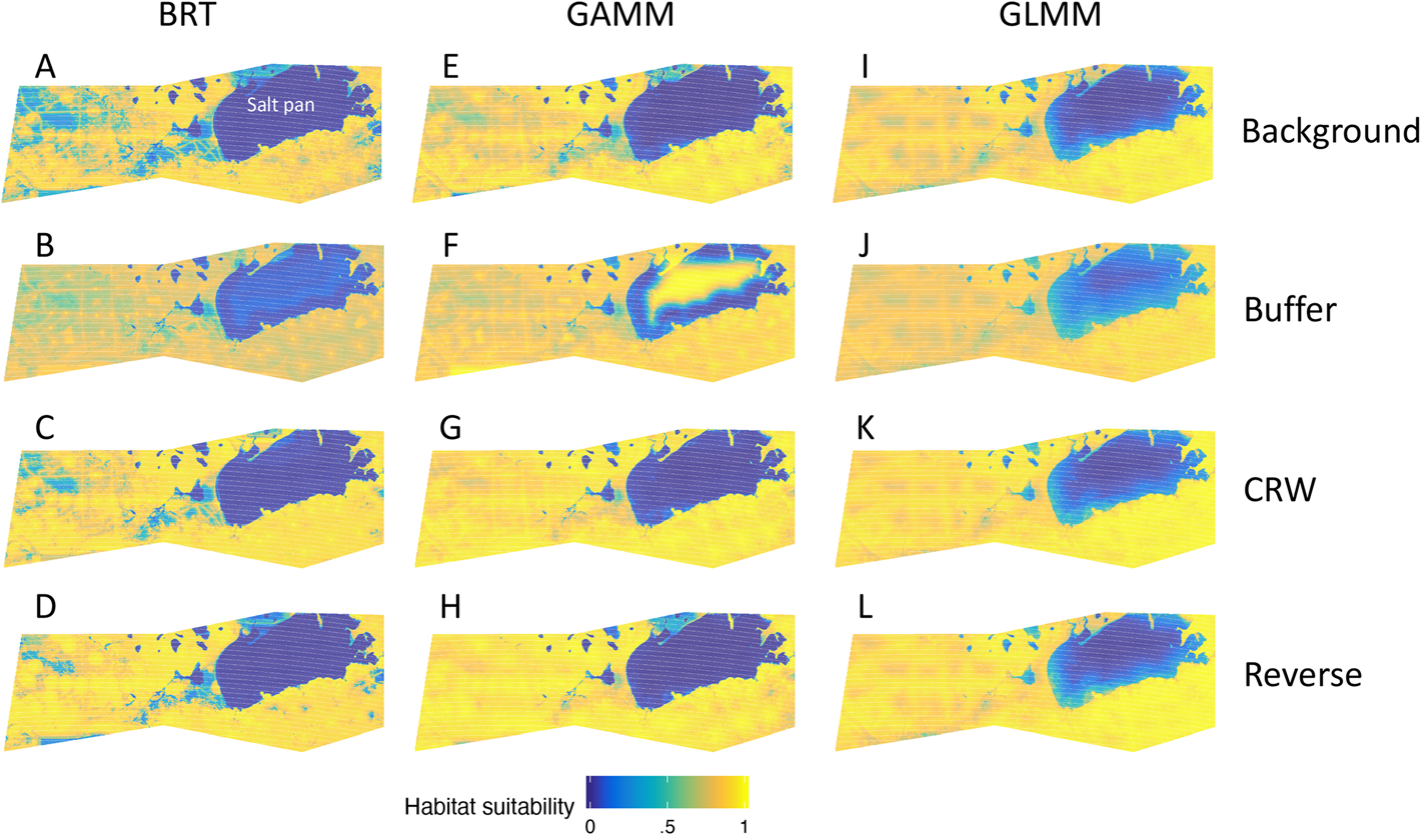
Effect of pseudo-absence generation type for elephants for BRT (**a**-**d**, four panels on left), GAMM (**e**-**h**), and GLMM models (**i**-**l**) and model type using background sampling (**a**, **e**, **i**, top three panels), buffer sampling (**b**, **f**, **j**), CRW sampling (**c**, **g**, **k**), and reverse CRW sampling (**d**, **h**, **l**) Yellow indicates high habitat suitability while blue is low habitat suitability

Spatial predictions of species distributions showed divergent results across pseudo-absence generations methods and model types. For blue whales, background sampling predicted more uniformly suitable habitat on the continental shelf, whereas other pseudo-absence methods predicted higher inshore use. CRWs and reverse CRWs were best able to reproduce the known blue whale hotspot in the Southern California Bight during summer months [12, 18, 36]. In general, there was more consistency in spatial predictions among model types than among pseudo-absence generation methods (Fig. 4). For elephants, spatial differences among both pseudo-absence methods and model types were minimal, with all (except GAMM with buffer) reproducing low habitat selection inside the large salt pan in the northeast of the park (Fig. 5). The BRT model with highest predictive skill was reverse CRW, while background sampling was able to highlight areas of low habitat preference in the northern extent of the EtNP that matched patterns in the tracking data to a greater degree than the other sampling methods and model types (Fig. 5). Elephant BRTs captured fine-scale patterns of habitat use across pseudo-absence types, while GLMMs and GAMMs predicted smoother and more homogeneous distributions (Fig. 5).

## Discussion

A critical component of habitat modeling for presence-only data like animal telemetry is selecting pseudo-absence points that provide insight into how habitat selected by animals differs from the range of available habitat [9]. Here we explored the performance of pseudo-absence generation techniques across species, study systems, and model types to help inform best practices for telemetry-based habitat modeling. We found that the environmental separation between presences and pseudo-absences was an important driver of model explanatory power and predictive skill - a result that held true across marine and terrestrial habitats, two species with different movement syndromes (migratory vs. nomadic), and three different model types. However, greater environmental separation between presences and pseudo-absences did not necessarily lead to greater biological realism in spatial predictions, highlighting the importance of using multiple inferences to evaluate model performance. Model performance metrics may be positively biased in cases where pseudo-absences are sampled from dissimilar habitats relative to those used by the study species, without a concurrent increase in the model’s ability to make accurate predictions of habitat use. This emphasizes the need to carefully consider the spatial extent of the sampling domain and environmental separation between presences and sampled pseudo-absences when developing habitat models.

Previous studies have demonstrated that model performance is influenced by study area extent and the proportion of this extent occupied by species, such that species that occupy small extents of a large study area are better predicted than species that occupy large extents of small study areas [44, 45, 62]. Separation in environmental niche space may dominate any differences between pseudo-absence generation approaches. For example, [51] found CRWs were less successful than background sampling. However, the study used CRWs only within the species’ domain and background sampling from outside the species’ domain to understand habitat use. Thus the separation between environmental conditions in the two sampling extents likely dominates any difference between pseudo-absence approach. Sampling across broad spatial and environmental gradients can be useful for identifying patterns of presence and absence and result in increased model performance, but may not be the most appropriate approach for understanding finer scale patterns of movement and habitat selection, highlighting the need to identify ecological questions and applications prior to modeling.

The four pseudo-absence methods differed in their ability to describe patterns in elephant and blue whale distributions, including correctly differentiating areas where species were probably present from areas where they were probably absent (e.g. offshore CCS, and in the Etosha salt pan). We assessed biological realism of our spatial predictions (Figs. 4 and 5) and found that the most biologically realistic models were not always those that performed best according to traditional model performance metrics. For example, blue whale background sampling had the highest predictive performance, but failed to identify the gradient between off-shelf absence and near-shore suitability where blue whales frequently occur. Background sampling tended to overestimate suitable habitat, and was therefore the most inaccurate at capturing areas where whales were absent (Table 1). In comparison, CRW sampling was more biologically realistic and better at capturing blue whale absence within the CCS domain despite this sampling approach resulting in models with poorer predictive performance and out of sample testing. Boosted regression tree models based on CRW and reverse CRW had anomalously high offshore habitat predictions where blue whales were rarely present even with strong realism nearshore, indicating these models would not be a good candidate for extrapolation [66].

The tradeoff between model skill and biological realism has practical implications for habitat modeling, where modellers should decide a priori on a model’s purpose and whether the ultimate goal is to better predict species presence or absence (e.g. [28]. We advise caution when comparing model performance across multiple studies that may be driven by different management goals or that use different underlying data, modeling types, or pseudo-absence generation approaches. For example, a blue whale habitat modeling application that aims to conservatively identify all areas where whales might be present in order to afford them maximum spatial protection could benefit from using the background method, whereas an application that seeks to identify areas where whales are most likely not in direct contact with human activities outside areas of core habitat use might benefit from the CRW approach. Ultimately, which pseudo-absence method is best for a given goal will depend to a large extent on what environmental range it is sampling compared to presences. Johnson [40] describes four orders of resource selection that animals may exhibit, ranging from coarse to fine spatial scales: a species’ geographic range (1st order); an area within the geographic range (e.g. a home range; 2nd order); an area within the home range (3rd order); and a specific site or resource within the selected area (4th order; [40]). We propose similar attention should be paid to the modeling or management aim to inform the pseudo-absence selection approach (see Table 2). Ultimately, ensemble approaches may be worth exploring to gain inference across model differences [4] or among data types and modeling approaches [65].

**Table 2:** Discussion of best practices for pseudo-absence selection method

**Scenario A:** Model purpose is to understand broadscale distribution of species habitat often averaged across multiple years [47, 57]. Background sampling has been used to understand where species could have been but were not sighted. These plots are useful for long-term planning and understanding general patterns of habitat use, for example planning military uses in the ocean, shipping lane designation, or off-shore energy sites. Based on Johnson [40] four orders of resource selection, background sampling can be targeted towards a species’ geographic range (1st order) or an area within the geographic range (e.g. a home range; 2nd order). Specific care needs to be taken to ensure that the background sampling extent represents the potential habitat and not beyond because oversampling can lead to inflated model skill. Background sampling often has the greatest environmental separation between presences and absences of the pseudo-absence methods explored.	
**Scenario B:** Model purpose is to describe fine-scale dynamic habitat of species [4, 30, 56, 67]. Correlated random walk sampling is used to create where an individual could have gone in the environment but did not choose to go. This approach is better at capturing fine scale changes in habitat as a function of changes in the environment, for example producing daily maps of predicted habitat to reduce bycatch, or ship-strike risk as a function of the changing environment. Reverse CRWs have also been used to counter the effects of tag-location bias on habitat selection [53]. CRW and reverse CRW both address Johnson [40] third-order of an area within the home range, and can be responsive towards more dynamic selection of habitat. These two approaches had intermediate separation between presences and absences of the pseudo-absence methods explored.	
**Scenario C:** Model purpose is to understand the factors that drive decision-making at each step for tagged individuals. Habitat models with buffer sampling are restricted to each location [19, 22]. Buffer pseudo-absence generation is used to assess individual potential steps rather than the track at a whole. This approach is best suited for understanding the fine-scale factors that influence habitat selection rather than broader habitat preferences, for example which habitat variables and anthropogenic features influence animal movements as they move through the landscape. Buffer sampling for species distribution models address similar aims as resource selection functions (RSF [16];) targeting Johnson [40] 4th order for specific site or resources within broader habitat. This method often results in the least environmental separations between presences and absences of the pseudo-absence methods explored.	

We found consistent rankings among the three habitat model types (GLMMs < GAMMs < BRTs) based on explanatory power and predictive skill. These patterns held across species despite differences among the pseudo-absence methods. For elephants in particular, model type had a larger impact on model results compared to the pseudo-absence method. This importance of model type for elephants may be a function of the static nature of the habitat model, where variation in elephant presence (locations every 6 h) was not as well explained by the environmental covariates and resulted in models with non-linear functions (BRTs and GAMMs) performing better than linear models (GLMMs). Further, the ability of BRTs to best predict elephant presence was likely a function of the sharp step-wise transitions in the response curves (e.g. recursive binary splits) that can best describe habitat preferences near discrete features such as water holes and roads.

Comprehensive comparisons of habitat model approaches exist elsewhere in the literature [11, 17, 23, 50], thus we explored the interaction between model type and pseudo-absence method to provide practical recommendations. We found that selection of the optimal pseudo-absence method varied based on the questions being asked of the model, on the animals’ movement syndromes [3], and on the width of environmental niche space sampled by presences and generated pseudo-absences. Single habitat models and single approaches towards model validation may be sufficient for exploring ecological inference, but when models are used for management or conservation purposes such as spatial planning, multiple approaches and validation metrics should be considered to ensure the robustness of design and implementation [4, 6, 48, 58]. Taken holistically, model purpose is of utmost importance when choosing pseudo-absence generation method and model type to ensure that predictions are tuned to scales of animal movement and management need.

## Conclusions

Maximizing predictive skill while maintaining biological realism is a key part of developing habitat models that optimize spatial protections for species while minimizing uncertainty and opportunity costs of erroneous predictions. Scientists have placed a lot of faith in quantitative metrics for evaluating predictive skill, but high performing models still may not be accurately addressing the research question at scale [27, 45]. Decisions such as choosing the most appropriate modeling framework for a given data structure and deciding how to represent absences can impact the robustness of models built for conservation and management applications. For this reason, careful consideration of model purpose and rigorous assessment of the robustness and accuracy of spatial predictions in relation to these decisions are important steps towards an improved understanding of the drivers of animal movement, predictions of habitat for use in spatial planning, and assessments of risk of human-wildlife conflicts.

## Supplementary Information

**Additional file 1: Table S1.** Summary of environmental predictors used in species distribution modelling for blue whale and elephant case studies. Spatial resolution is in decimal degrees. **Table S2.** Validation metrics for spatial and temporal hold-out approaches. Temporal hold-out not available for the average elephant model as predictor variables are not dynamically measured. **Figure S1.** Partial response curves for the two most important covariates in the blue whale (a-d) and elephant (e-h) habitat suitability models. Response curves for GAMMS (left panels; A, B, E, F), and BRTs (right panels; C, D, G, H) are shown for the four pseudo-absences generation techniques (Background in red, Buffer in gray, CRW in green, Reverse CRW in blue).

## Data Availability

The animal movement datasets used in the current study have been previously published and are available from the corresponding data holders (Bailey, Tsalyuk) on reasonable request. Blue whale data are also available on gtopp.org with elephant data available via Movebank: 10.5441/001/1.3nj3qj45. All code for use by this paper is available at https://github.com/elhazen/PA-paper, 10.5281/zenodo.4453501.

## Abbreviations

AUC: Area Under the receiver operating characteristic Curve
BRT: Boosted Regression Tree
CCS: California Current System
CRW: Correlated random walk
EtNP: Etosha National Park
GAMM: Generalised Additive Mixed Model
GLMM: Generalised Linear Mixed Model
TSS: True Skill Statistic
