# Supplementary material for: Where did they not go? Considerations for generating pseudo-absences for telemetry-based habitat models
Source: Mov Ecol. 2021 Feb 17;9:5. doi: 10.1186/s40462-021-00240-2 (PMC7888118; doi:10.1186/s40462-021-00240-2)
Supplement: Supplementary file 1 — Additional file 1: Table S1. Summary of environmental predictors used in species distribution modelling for blue whale and elephant case studies. Spatial resolution is in decimal degrees. Table S2. Validation metrics for spatial and temporal hold-out approaches. Temporal hold-out not available for the average elephant model as predictor variables are not dynamically measured. Figure S1. Partial response curves for the two most important covariates in the blue whale (a-d) and elephant (e-h) habitat suitability models. Response curves for GAMMS (left panels; A, B, E, F), and BRTs (right panels; C, D, G, H) are shown for the four pseudo-absences generation techniques (Background in red, Buffer in gray, CRW in green, Reverse CRW in blue). [file 40462_2021_240_MOESM1_ESM.docx]

**Supplementary Tables and Figures**

**Table S1** Summary of environmental predictors used in species distribution modelling for blue whale and elephant case studies. Spatial resolution is in decimal degrees.

**Table S2.** Validation metrics for spatial and temporal hold-out approaches. Temporal hold-out not available for the average elephant model as predictor variables are not dynamically measured.

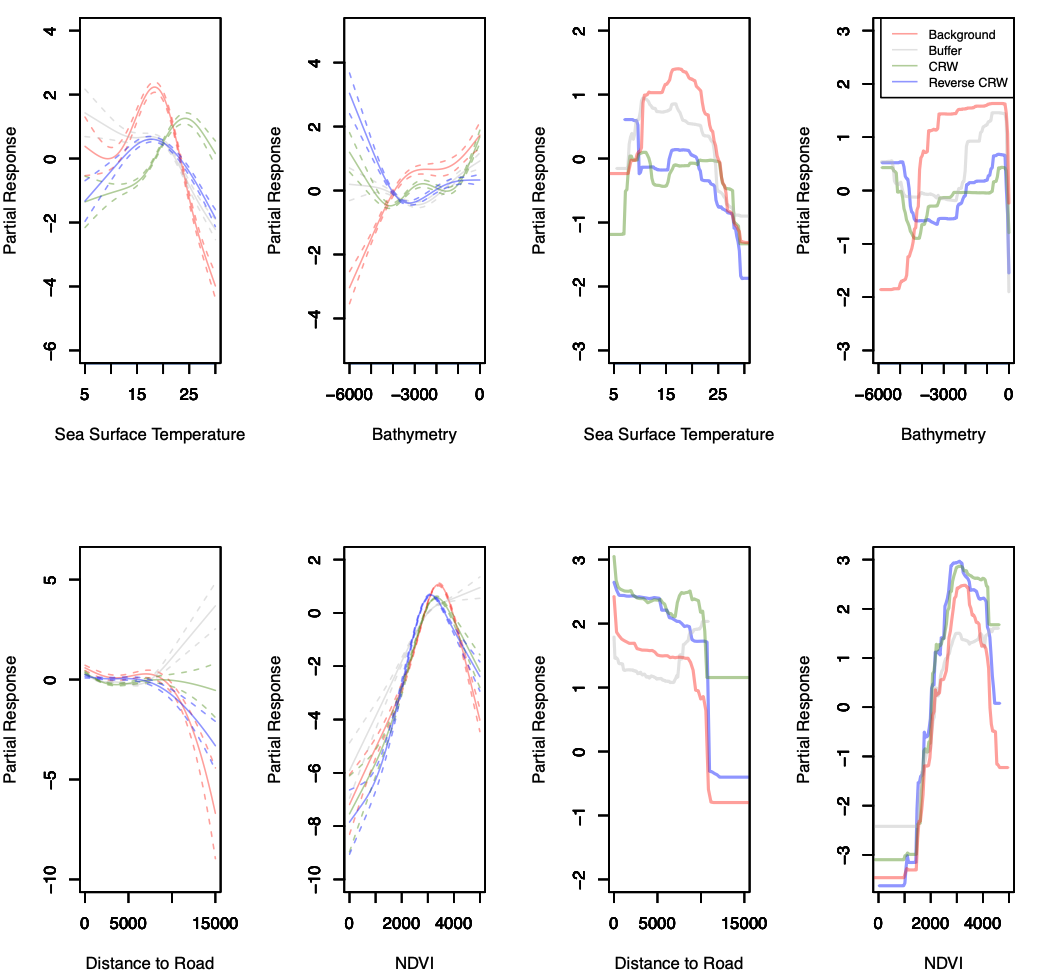
**Figure S1** Partial response curves for the two most important covariates in the blue whale (a-d) and elephant (e-h) habitat suitability models. Response curves for GAMMS (left panels; A,B,E,F), and BRTs (right panels; C,D,G,H) are shown for the four pseudo-absences generation techniques (Background in red, Buffer in gray, CRW in green, Reverse CRW in blue).

D

H

G

F

E

A

B

C
